# Effect of *Trichoderma reesei* Degraded Date Pits on Antioxidant Enzyme Activities and Biochemical Responses of Broiler Chickens

**DOI:** 10.3389/fvets.2020.00338

**Published:** 2020-08-18

**Authors:** Salem R. Alyileili, Khaled A. El-Tarabily, Ibrahim E. H. Belal, Wissam H. Ibrahim, Mohsin Sulaiman, Ahmed S. Hussein

**Affiliations:** ^1^Department of Integrative Agriculture, College of Food and Agriculture, United Arab Emirates University, Al-Ain, United Arab Emirates; ^2^Department of Biology, College of Science, United Arab Emirates University, Al-Ain, United Arab Emirates; ^3^Khalifa Center for Genetic Engineering and Biotechnology, United Arab Emirates University, Al-Ain, United Arab Emirates; ^4^College of Science, Health, Engineering and Education, Murdoch University, Murdoch, WA, Australia; ^5^Department of Food, Nutrition and Health, College of Food and Agriculture, United Arab Emirates University, Al-Ain, United Arab Emirates

**Keywords:** antioxidant indices, blood biochemistry, broiler chickens, degraded date pits, mannan-oligosaccharides, mannose, poultry nutrition

## Abstract

The long-term use of antimicrobials as growth promoters in poultry feed leads to antimicrobial resistance in pathogens. Thus, alternatives to antibiotics are essential for reasons associated with both safety and cost-effectiveness. Underutilized plant sources need to be developed to replace antibiotics in broiler feed. Several feed resources have been introduced so far, but they have yet to be applied widely. Date pits are a major by-product of the date industry (6–8%) and have the potential antioxidant to replace antibiotics. In this study, fresh date pits were degraded using the mold *Trichoderma reesei* under solid-state degradation (SSD), resulting in degraded date pits (DDP). A total of 180 Brazilian “Cobb 500” broiler chicks were divided into six feed treatments in triplicate groups. The treatments were corn-soy basal diet (positive control; C+), corn-soy + 20% oxytetracycline at 0.05% (negative control; C–), corn-soy + 10% DDP, corn-soy + 0.2% mannan-oligosaccharides (MOS), corn-soy + 0.1% mannose, and corn-soy + 0.2% mannose. The antioxidant and biochemical effects of DDP, MOS, and mannose were determined in the blood serum, liver, and intestine of broilers at age 21 and 42 days. The results indicated that the contents of antioxidants such as flavonoids and phenolics, as well as the MOS content in DDP, were increased by the degradation process. Additionally, mannose, glucose, arabinose, rhamnose, and glucuronic acid were significantly increased in DDP after degradation. The activity of antioxidant enzymes (GPx—glutathione peroxidase, catalase, and SOD—superoxide dismutase) in the serum, liver, and intestine of broilers fed with diets containing 10% DDP and 0.2% MOS was increased significantly compared to the control group. Malondialdehyde activity was decreased, whereas the mean corpuscular hemoglobin level and the iron content were significantly upregulated in the broilers fed with 10% DDP, 0.1% mannose, and 0.2% MOS diets compared with the control. Thus, DDP can be used to improve the antioxidant status and has a prebiotic-like effect in broiler chicken performance.

## Introduction

Practical applications of agricultural wastes in poultry nutrition play an important role in replacing expensive ingredients and/or additives in their diet particularly after COVID-19 outbreaks in which an expected shortage of feed resources for animal nutrition may have occurred (1–3). Poultry are a valuable source of animal protein of high quality, and nutrition of poultry plays an essential role in controlling the balance of pro-oxidants and antioxidants and thus product quality and shelf life after harvest (4).

The synthesis of reactive oxygen species (ROS) affects the antioxidant system and pro-oxidants, leading to damage, which results in oxidative stress (5). Oxidative stress disrupts redox signaling, and thus measuring the redox ratio is an effective method for studying oxidative stress. Broilers are vulnerable to different types of stressors, such as the stress caused by heat, which induces the generation of ROS. Currently, consideration is being given to new methods of protection against ROS using antioxidant enzymes.

Antioxidant enzymes are present in all organisms, and these enzymes help prevent cell membrane damage, the inactivation of enzymes, and alteration of nucleic acids. The major enzymes that make up the primary defenses are glutathione peroxidase (GPx), catalase (CAT), and superoxide dismutase (SOD). SOD catalyzes the dismutation of superoxide radicals to H_2_O_2_ and oxygen, while CAT catalyzes the breakdown of H_2_O_2_ to H_2_O and molecular oxygen. GPx is a selenium-based enzyme, which deactivates peroxides using the peptide glutathione (GSH) as its cosubstrate (6). Catalase and peroxidases are enzymatic ROS scavengers that decrease the concentration of H_2_O_2_, which acts as a source of active radical species. ROS are deemed as critical oxygen mediators and crucial messengers that promote cell division (7). The lipid peroxidation end product malondialdehyde (MDA) in tissues acts as a biomarker for radical-prompted deterioration and peroxidation of endogenous lipid.

The catalytic activity of enzymes in erythrocytes and the liver is most commonly monitored for the diagnosis of blood and organ diseases. Erythrocytes are rich in hemoglobin with an efficient system of defense against free radicals, and they contain antioxidant enzymes with a high level of glutathione (8). The liver performs primary detoxification functions and has a central metabolic role in the organism (9). Antioxidant enzymes neutralize the formation and harmful effects of reactive oxygen metabolites (10).

Date palm (*Phoenix dactylifera* L.) is one of the major fruit crops in most of the Arabian countries. Solid-state degradation (SSD) of date pits with exogenous microbial enzymes like xylanases enhances the production of simpler forms of carbohydrate molecules from fibers present in date pits (11). A considerable investigation is currently concentrated on the cellulolytic filamentous fungus, *Trichoderma reesei*. When subjected to degradation, enzymes secreted by *T. reesei* catalyze the degeneration of these substrates to simple sugars and enhance the degradation of plant cell walls. Degradation of date pits improves the chemical constituents and yields bioactive substances of added value for animal nutrition (1–3), and SSD is one of the reasonable methods to prepare degraded date pits (DDP) by using *T. reesei*.

Microbially degraded feeds and enzymes can be better utilized by animals, which improve the growth performance and protection in stress condition and maintain productive and reproductive performance (12). The advantage of using enzymatically degraded prebiotics like DDP in animal feed promotes the beneficial role of non-digestible sugars for increasing the immune system of animals through the antioxidant system. This property of a natural growth promoter from date pits is a milestone to animal feed research. Thus, the goal of the current study was to investigate the impacts of DDP, mannan-oligosaccharides (MOS), and mannose on the blood serum, liver, and intestine antioxidant and the biochemical responses of broiler chickens.

## Materials and Methods

The scientific committee of the Department of Integrative Agriculture, College of Food and Agriculture, United Arab Emirates University, United Arab Emirates (UAE) approved the present work under experimental protocol 313072. The committee recommended animal welfare and minimum stress during the experimental work.

### Date Pits

Fresh pits of dates (*P. dactylifera* L.), Khalas variety, were acquired from the Al Sad date processing factory in Al-Ain, UAE. Each seed was ~2–2.5 cm long and ~6–8 mm thick. A medium-sized mill (Skiold A/S, Kjeldgaardsvej 3, Saeby 9300, Denmark) was used to finely grind the date pits to about 1 mm in diameter.

### Preparation of *T. reesei* Culture

*T. reesei* used in the current study was cultivated as described by Hussein et al. (13). Four lyophilized *T. reesei* ampoules were purchased from Deutsche Sammlung von Mikroorganismen und Zellkulturen GmbH (DSMZ), Braunschweig, Germany. A subsample from the rehydrated *T. reesei* culture was transferred to potato dextrose broth (PDB) (Lab M Limited, Lancashire, UK) amended with 250 μg ml^−1^ chloramphenicol (Sigma-Aldrich Chemie GmbH, Taufkirchen, Germany) and 100 μg ml^−1^ streptomycin sulfate (Sigma-Aldrich). A rotary shaker (Model G76, New Brunswick Scientific, Edison, NJ, USA) was used for the incubation of the flasks at 250 rpm at 25 ± 2°C in the dark for 7 days. The flasks were assessed visually daily to monitor the growth of fungus. The fungus was kept on PDA plates and stored at 4°C.

### Preparation of *T. reesei* DDP and the Experimental Diets

*T. reesei* DDP was produced using an SSD inside an incubator as described by Hussein et al. (13). Chemical composition of DDP and non-degraded date pits (NDDP) (*n* = 3 per treatment) was studied and reported by Alyileili et al. (14). Diets were formulated (Table 1) to be isonitrogenous and isocaloric. The calculated nutrient composition of the diets was based on the feedstuff profiles reported by Hashim et al. (15). All ingredients were ground and mixed in a commercial mixer (Hobart mixer, HL1400, USA) for 20 min.

**Table 1:** Composition of experimental finisher diets on dry matter basis for broilers.

**Ingredients, %**	**1**	**2**	**3**	**4**	**5**	**6**
Yellow corn	64.6	64.6	52.1	64.4	64.4	64.5
Soybean meal	28.4	28.4	26.0	28.4	28.4	28.4
Salt	0.42	0.42	0.33	0.42	0.42	0.42
Limestone	1.33	1.28	1.19	1.33	1.33	1.33
Dicalcium phosphate	1.05	1.05	0.8	1.05	1.05	1.05
Vit + Min. Premix^a^	0.2	0.2	0.2	0.2	0.2	0.2
DL-Methionine	0.2	0.2	0.3	0.2	0.2	0.2
Lysine	0.1	0.1	0.18	0.1	0.1	0.1
Corn oil	2.5	2.5	5.9	2.5	2.5	2.5
Fish meal	1.2	1.2	3.0	1.2	1.2	1.2
Degraded date pits	0	0	10	0	0	0
Oxytetracycline	0	0.05	0	0.0	0.0	0.0
Mannan-oligosaccharides	0	0	0	0.2	0	0
Mannose	0	0	0	0	0.2	0.1
Total	100	100	100	100	100	100
Calculated and determined chemical composition (% of DM basis)						
ME, MJ/kg diet	13.12	13.12	13.12	13.12	13.12	13.12
Crude protein	17.8	17.6	17.7	17.8	17.8	17.8
Methionine	0.47	0.47	0.49	0.47	0.47	0.47
Methionine + cysteine	0.86	0.86	0.84	0.86	0.86	0.86
Lysine	1.05	1.05	1.10	1.05	1.05	1.05
Threonine	0.71	0.71	0.73	0.71	0.71	0.71
Crude fiber	3.09	3.09	3.92	3.09	3.09	3.09
Calcium	0.82	0.82	0.80	0.82	0.82	0.82
Available phosphorus	0.39	0.39	0.38	0.39	0.39	0.39
Sodium	0.20	0.20	0.20	0.20	0.20	0.20

*DM, Dry matter*;

a *Supplementary levels of vitamins and trace elements (per kg): Vitamin A, 5484 IU; Vitamin D_3_, 2822 ICU; Vitamin E (as DL alpha-tocopherol acetate) 26 IU; Vitamin K (as menadione sodium bisulfite), 4.38 mg; thiamine, 5.94 mg; Riboflavin, 6.2 mg; Pyridoxine, 4.5 mg; Cyanocoblamine, 0.14 mg; Niacin (as nicotinic acid), 44.1 mg; D-pantothenic acid, 15 mg; Folic acid, 990 μg; Biotin, 0.23 mg; Iron, 120 mg; Copper, 8 mg; Manganese, 83 mg; Cobalt, 5 mg; Zinc, 60 mg; Iodine, 1.11 mg; Selenium, 300 μg. 1. corn soybean meal diet (Control +); 2. corn soybean meal diet + antibiotic (Control −); 3. corn soybean meal diet + 10% degraded date pits; 4. corn soybean meal diet + 0.2% mannan-oligosaccharides; 5. corn soybean meal diet + 0.2% Mannose; 6. corn soybean meal diet + 0.1% Mannose*.

A total of 180 Brazilian “Cobb 500” broiler chicks were divided into six dietary treatments. The treatments were T1—corn-soy basal diet (Positive control; C+), T2—corn-soy basal diet + 20% oxytetracycline at 0.05% (Negative control; C–), T3—corn-soy basal diet + 10% (DDP), T4—corn-soy basal diet + 0.2% (MOS), T5—corn-soy basal diet + 0.1% mannose, and T6—corn-soy basal diet + 0.2% mannose. DDP was given simultaneously with oxytetracycline to study the antibacterial effect of DDP in broiler intestine. Chickens were housed as 10 chicks per cage (50 × 45 × 45 cm) in an environmentally controlled house.

The experiment lasted for 42 days from 1 to 42 days of age. Birds within each treatment were designated to three replicate groups (*n* = 10/group). Feed and water were given on an *ad libitum* basis. The light-dark cycle was 23:1 daily from the 4 days of the experiment. Vaccination and medical care were carried out under the supervision of veterinaries.

Chicken liver and intestinal and blood samples (serum and plasma) were collected in two tubes with or without heparin from two randomly selected broilers at 42 days of age from each treatment replicate (*n* = 6 samples). Blood samples were centrifuged at 1,716 g for 15 min to separate the plasma and serum, which were kept at 20°C until analysis.

### Mannan-Oligosaccharides (MOS) and Antioxidant Agents From Degraded and Non-degraded Date Pits (NDDP)

Oligosaccharides were extracted from DDP and NDDP using the method of Huang et al. (16). Extracted sample was purified, and monosaccharide composition was determined by the method of Jahromi et al. (17). In DDP and NDDP (*n* = 3 per treatment), the ability to scavenge 2,2-diphenyl-1-picrylhydrazyl (DPPH) free radicals was assayed according to a previously described procedure (18). The 2,2-azino-bis-3-ethylbenzothiazoline-6-sulfonic acid (ABTS) radical scavenging was measured using another previously described procedure (19). The ferric reducing activity of the date pits extract was assessed based on the method reported by Benzie and Strain (20).

### Biochemical Constituents

Malondialdehyde (MDA) was measured in liver and intestinal tissue and in blood serum using the thiobarbituric acid assay method as described by Ohkawa et al. (21). Catalase activity was assayed in liver and intestinal tissue and blood serum as described by Maehly (22). SOD activity was measured in liver and intestinal tissue and blood serum as described by Kakkar et al. (23). The activity of GPx was evaluated in the liver as described by Lawrence and Burk (24) with modifications described by Agergaard and Jensen (25). Alanine aminotransferase (ALT), aspartate aminotransferase (AST), and glutamate-pyruvate transaminase were evaluated in serum using the procedure described by Rietman and Frankel (26). Gamma glutamyl transferase (GGT) activity was measured using a GGT colorimetric assay kit from Sigma-Aldrich. The total calcium, iron, phosphorus, and copper in plasma were quantified by inductively coupled atomic emission spectrometry (ICP-OES), as described by Vanhoe et al. (27). Blood serum urea was measured using the diacetyl monoxime method (28). Blood serum uric acid was estimated using the modified colorimetric technique (29). Total protein, cholesterol, blood plasma glucose, creatinine, triglycerides, and HDL-cholesterol were quantified using commercial kits (Unichem Elite, United Diagnostics Industry, Dammam, KSA) and based on the methods used by Piotrowska et al. (30).

### Hematological Parameters

The hemocytometer method using the Natt-Herrick solution was used for the determination of white blood cell (WBC), red blood cell (RBC), and hemoglobin (Hb) count, and hematocrit values were measured using the microhematocrit and cyanate-hemoglobin method (31). The mean corpuscular volume (MCV), mean corpuscular hemoglobin (MCH), and mean corpuscular hemoglobin concentration (MCHC) were measured as described by Pampori and Iqbal (32).

### Statistical Analysis

Data were subjected to ANOVA (one-way procedure) using a general linear model (GLM), and mean comparisons were done using the Student Newman Keuls test to compare significant differences among means for all analyses (Version 20.0, SPSS Inc., Chicago, IL, USA). The differences among means were considered significant at *p* ≤ 0.05.

The statistical model was as follows:

Model:

(1) $$\begin{array}{r} X_{ij}=u+\;T_{i\;}+e_{ij}\; \end{array}$$

where

*X*_*ij*_ = *Any observation*

*u* = *Overall mean*

*T*_*i*_ = *Treatments* (*i* = 1, 2…*and* 4)

*e*_*ij*_ = *Experimental error*

The number of samples used in the statistical analyses was six per treatment as two samples per replicate considering the sample as the experimental unit. This was done to improve the precision of analyses of variance. Before analyses of variance, all percentage data were transformed to their analogous arcsine.

## Results

### Phenolic, Flavonoid, and Antioxidant Activity of DDP and NDDP

The phenolic content in NDDP was 3.2 g GAE/100 g DW, and the flavonoid content was 2.28 g RE/100 g DW. After degradation with *T. reesei*, the flavonoid and phenolic contents were significantly increased to 11.68 g RE/100 g DW and 14.23 g GAE/100 g DW, respectively. These increases amounted to 11.03 and 9.6 g, respectively (Table 2).

**Table 2:** Phenolic, flavonoid content, and antioxidant activities of date pits (Means ± SE; *n* = 3).

**Parameters**	**Non-degraded date pits, %**	**Degraded date pits,%**
Total phenol, g GAE/100 g DW^a^	3.21 ± 0.712	14.2 ± 1.29*
Flavonoids, RE/100 g DW^b^	2.28 ± 0.850	11.7 ± 2.21*
DPPH activity, %^c^	59 ± 9.2	78 ± 11.9*
ABTS, mmol TE/100 g DW3^d^	6.23 ± 0.361	13.3 ± 1.89*
FRAP, mmol TE/100 g DW^d^	24.6 ± 4.58	36.2 ± 6.39*
Metal chelating activity, μmol EE /g DW^e^	13.3 ± 0.221	17.3 ± 2.681

*SE, standard error*.

* *P < 0.05*.

a *mg GAE/100 g DW, mg Gallic acid equivalent per 100 g dry weight of date pits*;

b *RE/100 g DW, Rutin equivalents per 100 g dry weight of date pits*;

c *DDPH, α, α-diphenyl-β-picrylhydrazyl*,

d *mmol TE/100 g DW, mmol Trolox equivalents per 100 g dry weight of date pits*;

e *μmol EE /g DW, μmol EDTA equivalents per g dry weight of date pits. 2,2-diphenyl-1-picrylhydrazyl (DPPH), 2,2-azino-bis-3-ethylbenzothiazoline-6-sulfonic acid (ABTS), Ferric reducing antioxidant power (FRAP)*.

These findings indicated that, after degradation with *T. reesei*, the DPPH radical scavenging ability was 78% in DDP compared to 59% in NDDP. This represents an increase of 19%. The ABTS radical scavenging activity of NDDP was 6.23 mmol TE/100 g DW, while it was significantly higher at 13.28 mmol TE/100 g DW in DDP. Additionally, the ferric reducing antioxidant power (FRAP) assay showed that the degradation process with *T. reesei* enhanced the ferric reducing antioxidant power of NDDP from 24.56 mmol TE/100 g DW to 36.23 mmol TE/100 g DW. Metal chelating activity was also increased in DDP to about 17.25 μmol EE/g DW. Table 3 shows the MOS content and its monosaccharide composition in NDDP. Degradation by *T. reesei* significantly increased the MOS content in date pits. The mannose content of MOS from DDP was 19.97%. After degradation, the contents of glucose, arabinose, rhamnose, and glucuronic acid were significantly increased in MOS.

**Table 3:** Monosaccharides contents in mannan-oligosaccharides of non-degraded and degraded date pits (Means ± SE; *n* = 3).

**Component**	**Non-degraded date pits, %**	**Degraded date pits, %**
Mannan-oligosaccharides	16.5 ± 3.01	30.0 ± 4.26*
Monosaccharides, % on dry matter basis		
Galactose	13.1 ± 0.601	22.3 ± 4.31
Mannose	5.94 ± 0.381	20.0 ± 1.70*
Glucose	6.97 ± 0.70	14.9 ± 1.51*
Arabinose	2.41 ± 0.301	4.91 ± 0.550*
Xylose	0.713 ± 0.041	5.56 ± 0.170
Rhamnose	3.01 ± 0.260	5.16 ± 0.680*
Glucuronic acid	15.0 ± 0.940	27.4 ± 3.01*

*SE, standard error*.

* *P < 0.05*.

### Effect of Treatments on Activity of Serum SOD, CAT, GPx, and MDA

The activity of the enzymatic antioxidants SOD, CAT, and GPx in serum was found to be significantly higher in broilers fed with the 10% DDP diet and the 0.2% MOS diet as compared to those fed with the corn-soy diet. The activity of the antioxidant enzymes in broilers fed with the antibiotic and mannose diets increased but was not significantly higher than that in broilers fed with the 10% DDP and 0.2% MOS diets (Table 4). The level of MDA was significantly lower in broilers fed with the 10% DDP diet and 0.2% MOS diet compared with those fed with the positive and negative control diets or the mannose diet.

**Table 4:** Effect of different dietary treatments on activity of SOD, CAT, GPx, and MDA in serum (Means ± SE; *n* = 6).

**Parameters**	**Dietary treatments**					
	**1**	**2**	**3**	**4**	**5**	**6**
SOD (U/mg)	127 ± 9.4^b^	161 ± 18.5^ab^	224 ± 17.0^a^	231 ± 20.1^a^	165 ± 16.1^ab^	166 ± 14.6^ab^
Catalase (U/mg)	29.6 ± 3.90^b^	46.8 ± 9.75^b^	91.0 ± 10.99^a^	87.9 ± 12.83^a^	60.5 ± 7.82^ab^	66.0 ± 9.05^ab^
GPx (U/mg)	216 ± 17.3^b^	230 ± 20.7^b^	313 ± 26.4^a^	319 ± 29.2^a^	215 ± 7.9^b^	219 ± 11.2^b^
MDA (nmol/ml)	5.97 ± 0.262^a^	5.81 ± 0.971^a^	1.94 ± 0.122^b^	1.76 ± 0.071^b^	4.96 ± 0.191^a^	4.12 ± 0.121^a^

*^a, b^Means within a row with different superscripts are significantly different (P < 0.05). SE, standard error; 1. corn soybean meal diet (Control +); 2. corn soybean meal diet + antibiotic (Control –); 3. corn soybean meal diet + 10% degraded date pits; 4. corn soybean meal diet + 0.2% mannan-oligosaccharides; 5. corn soybean meal diet + 0.2% Mannose; 6. corn soybean meal diet + 0.1% Mannose. SOD, superoxide dismutase; CAT, catalase; GPx, glutathione peroxidase; MDA, malondialdehyde*.

### Effect of Treatments on Activity of SOD, CAT, GPx, GST, and MDA in Liver

The activity of SOD and CAT in the liver was significantly higher in broilers fed with the 10% DDP diet and 0.2% MOS diet compared with those fed with the positive control diet (Table 5). The activity of SOD was similar in mannose and negative control fed-broilers and did not differ significantly from the other groups. The results showed that CAT activity was intermediate in the broilers fed with the positive control diet and did not differ significantly from that in the other groups. The GPx activity was similar in broilers fed with the 0.1 and 0.2% mannose diets and was comparable to that in broilers fed with the 10% DDP and 0.2% MOS diets. The GPx activity in the liver was significantly higher in broilers fed with the 10% DDP diet and 0.2% MOS diet compared with those fed with the positive control diet or 0.2% mannose. The liver glutathione S-transferase (GST) activity was significantly higher in broilers fed with the 10% DDP and 0.2% MOS diets when compared with those fed with the positive control diet. Additionally, the GST activity of broilers fed with the 0.1% mannose and negative control diets was significantly higher than in those fed with the positive control diet, but lower than in those fed with the DDP and MOS diets (Table 5). The liver MDA content was significantly lower in broilers fed with the 10% DDP and 0.2% MOS diets when compared with those fed with the positive control diet. The MDA content in broilers fed with the mannose and positive and negative control diet was similar and significantly higher than that in those fed with other test diets.

**Table 5:** Effect of different dietary treatments on activity of SOD, CAT, GPx, GST, and MDA content in liver (Means ± SE; *n* = 6).

**Parameters**	**Dietary treatments**					
	**1**	**2**	**3**	**4**	**5**	**6**
SOD (U/mg)	0.801 ± 0.111^b^	1.26 ± 0.191^ab^	1.61 ± 0.151^a^	1.57 ± 0.052^a^	1.33 ± 0.131^ab^	1.27 ± 0.032^ab^
Catalase (U/mg)	2.83 ± 0.381^b^	4.23 ± 0.670^ab^	5.48 ± 0.421^a^	5.54 ± 0.301^a^	4.19 ± 0.320^ab^	4.88 ± 0.420^ab^
GPx (U/mg)	0.290 ± 0.061^b^	0.491 ± 0.041^ab^	0.561 ± 0.020^a^	0.571 ± 0.020^a^	0.341 ± 0.021^b^	0.421 ± 0.031^ab^
GST (U/mg)	1.24 ± 0.061^c^	1.46 ± 0.101^b^	1.80 ± 0.041^a^	1.74 ± 0.031^a^	1.41 ± 0.010^ab^	1.58 ± 0.021^b^
MDA (nmol/ml)	3.12 ± 0.270^a^	2.88 ± 0.220^a^	1.99 ± 0.272^b^	1.85 ± 0.070^b^	2.56 ± 0.151^a^	2.45 ± 0.370^a^

*^a, b and c^ Means within a row with different superscripts are significantly different (P < 0.05). SE, standard error; 1. corn soybean meal diet (Control +); 2. corn soybean meal diet + antibiotic (Control–); 3. corn soybean meal diet + 10% degraded date pits; 4. corn soybean meal diet + 0.2% mannan-oligosaccharides; 5. corn soybean meal diet + 0.2% Mannose; 6. corn soybean meal diet + 0.1% Mannose. SOD, superoxide dismutase; CAT, catalase; GPx, glutathione peroxidase; GST, glutathione S-transferase; MDA, malondialdehyde*.

### Effect of Treatments on Activity of SOD, CAT, GPx, GST, and MDA Content in Intestine

The intestinal SOD, CAT, GPx, and GST activity was significantly higher in broilers fed with the 10% DDP diet and 0.2% MOS diet in both the proximal and distal portions compared with broilers fed with the positive control diet. The SOD activity of broilers fed with the mannose diet in the proximal and distal portions of the intestine was lower when compared with those fed with the 10% DDP and 0.2% MOS diets. In broilers fed with the mannose diets, the activity of CAT and GPx in the proximal and distal portions of the intestine was similar to that in broilers fed with positive and negative control diets. The activity of GST in the proximal portion of the intestine of broilers fed with the mannose diet was low and comparable to that in broilers fed with positive and negative control diets. The MDA content in the proximal and distal portions of the intestine was significantly lower in broilers fed with the 10% DDP and 0.2% MOS diets compared with those fed with the positive control diet. The MDA content in both portions of the intestine in broilers fed with the mannose diet was comparable with that in broilers fed with positive and negative control diets (Table 6).

**Table 6:** Effect of different dietary treatments on activity of SOD, CAT, GPx, GST, and MDA content in intestine (Means ± SE; *n* = 6).

**Parameters**	**Dietary treatments**											
	**1**		**2**		**3**		**4**		**5**		**6**	
	**Proximal**	**Distal**	**Proximal**	**Distal**	**Proximal**	**Distal**	**Proximal**	**Distal**	**Proximal**	**Distal**	**Proximal**	**Distal**
SOD (U/mg)	0.750 ± 0.041^b^	0.821 ± 0.040^b^	0.980 ± 0.060^b^	1.10 ± 0.081^b^	1.37 ± 0.091^a^	1.35 ± 0.091^a^	1.28 ± 0.031^a^	1.54 ± 0.061^a^	0.810 ± 0.701^b^	0.951 ± 0.05^b^	0.881 ± 0.041^b^	1.24 ± 0.091^b^
CAT (U/mg)	0.1000 ± 0.000^b^	0.750 ± 0.000^b^	0.350 ± 0.061^b^	1.23 ± 0.100^b^	0.530 ± 0.040^a^	1.53 ± 0.080^a^	0.601 ± 0.080^a^	1.67 ± 0.060^a^	0.410 ± 0.070^ab^	1.18 ± 0.07^b^	0.450 ± 0.091^ab^	1.15 ± 0.090^b^
GPx (U/mg)	0.380 ± 0.031^b^	1.00 ± 0.060^b^	0.620 ± 0.000^ab^	1.31 ± 0.060^ab^	1.19 ± 0.101^a^	1.73 ± 0.120^a^	1.28 ± 0.040^a^	1.49 ± 0.070^a^	0.891 ± 0.071^ab^	1.12 ± 0.05^ab^	0.810 ± 0.082^ab^	1.36 ± 0.100^ab^
GST (U/mg)	0.051 ± 0.000^b^	0.021 ± 0.000^b^	0.061 ± 0.000^b^	0.051 ± 0.000^ab^	0.08 ± 0.000^a^	0.071 ± 0.000^a^	0.07 ± 0.000^a^	0.06 ± 0.000^a^	0.060 ± 0.000^b^	0.041 ± 0.00^ab^	0.061 ± 0.001^b^	0.04 ± 0.001^ab^
MDA (nmol/ml)	8.94 ± 1.14^b^	10.3 ± 0.440^b^	6.68 ± 0.921^ab^	6.12 ± 0.320^ab^	4.95 ± 0.861^a^	4.02 ± 0.480^a^	4.58 ± 0.700^a^	4.12 ± 0.391^a^	7.23 ± 0.670^ab^	7.42 ± 0.56^ab^	7.23 ± 0.671^ab^	7.60 ± 0.481^ab^

*^a, b^Means within a row with different superscripts are significantly different (P < 0.05). SE, standard error. 1. corn soybean meal diet (Control +); 2. corn soybean meal diet + antibiotic (Control–); 3. corn soybean meal diet + 10% degraded date pits; 4. corn soybean meal diet + 0.2% mannan-oligosaccharides; 5. corn soybean meal diet + 0.2% Mannose; 6. corn soybean meal diet + 0.1% Mannose. SOD, superoxide dismutase; CAT, catalase; GPx, glutathione peroxidase; GST, glutathione S-transferase; MDA, malondialdehyde*.

### Effect of Treatments on Blood Biochemistry

The supplementation of 10% DDP, 0.2% MOS, and 0.1% or 0.2% mannose in broiler diets had no influence on ALT, AST, GGT, plasma glucose, calcium, creatinine, copper, phosphorus, or uric acid. Triglycerides, total cholesterol, LDL cholesterol, and HDL cholesterol were reduced in broilers fed with the 10% DDP and 0.2% MOS diets, but not significantly compared with the other treatments. The iron content was significantly higher in broilers fed with 10% DDP, 0.2% mannose, and 0.2% MOS when compared with broilers fed with the positive and negative control diets (Table 7).

**Table 7:** Effect of different dietary treatments on blood biochemical parameters of broilers (Means ± SE; *n* = 6).

	**Dietary treatments**					
**Parameters**	**1**	**2**	**3**	**4**	**5**	**6**
Glucose, mg/dl	262 ± 2.9^a^	271 ± 3.5^a^	270 ± 4.9^a^	265 ± 5.0^a^	268 ± 5.3^a^	274 ± 4.3^a^
Total cholesterol, mg/dl	161 ± 3.7^a^	167 ± 7.9^a^	152 ± 7.8^a^	157 ± 8.9^a^	155 ± 8.7^a^	164 ± 2.2^a^
Triglyceride, mg/dl	92.5 ± 5.21^a^	99.3 ± 6.87^a^	86.3 ± 5.73^a^	89.3 ± 4.26^a^	91.6 ± 3.89^a^	91.1 ± 4.60^a^
LDL cholesterol, mg/dl	50.0 ± 5.98^a^	60.2 ± 3.42^a^	47.3 ± 8.79^a^	44.5 ± 5.98^a^	41.6 ± 5.60^a^	42.9 ± 5.34^a^
HDL cholesterol, mg/dl	98.7 ± 0.48^a^	95.9 ± 1.02^a^	91.2 ± 4.51^a^	93.1 ± 1.27^a^	94.4 ± 2.68^a^	96.5 ± 6.74^a^
Creatinine, mg/dl	0.411 ± 0.005^a^	0.421 ± 0.005^a^	0.411 ± 0.005^a^	0.363 ± 0.012^a^	0.424 ± 0.005^a^	0.442 ± 0.017^a^
Total protein, g/dl	3.43 ± 0.170^a^	3.54 ± 0.201^a^	3.50 ± 0.161^a^	3.59 ± 0.220^a^	3.66 ± 0.241^a^	4.06 ± 0.091^a^
ALT, IU/l	5.23 ± 0.510^a^	5.32 ± 0.530^a^	5.27 ± 0.511^a^	5.27 ± 0.490^a^	5.26 ± 0.550^a^	5.63 ± 0.731^a^
AST, IU/l	181 ± 13.9^a^	194 ± 14.1^a^	184 ± 12.6^a^	179 ± 13.2^a^	183.4 ± 25.9^a^	185.4 ± 4.1^a^
GGT, IU/l	37.7 ± 2.59^a^	40.5 ± 3.47^a^	38.6 ± 1.79^a^	33.0 ± 2.06^a^	36.5 ± 3.18^a^	42.3 ± 4.62^a^
Calcium, mg/dl	16.8 ± 1.65^a^	17.9 ± 2.21^a^	19.4 ± 2.03^a^	17.7 ± 1.89^a^	18.9 ± 1.73^a^	15.5 ± 1.67^a^
Phosphorus, mg/dl	5.33 ± 0.870^a^	5.13 ± 1.131^a^	6.81 ± 1.401^a^	5.49 ± 0.620^a^	5.79 ± 1.291^a^	6.43 ± 0.670^a^
Iron, μg/dl	107 ± 6.06^b^	100 ± 1.92^b^	122 ± 1.93^a^	113 ± 5.04^a^	117 ± 4.44^a^	118 ± 2.12^a^
Copper, μg/dl	39.6 ± 2.59^a^	34.8 ± 2.03^a^	41.5 ± 1.67^a^	41.7 ± 2.42^a^	38.6 ± 2.25^a^	34.4 ± 2.78^a^
Urea, mg/dl	5.53 ± 0.391^a^	5.97 ± 1.541^a^	3.37 ± 0.332^a^	3.59 ± 0.452^a^	4.59 ± 0.551^a^	3.53 ± 0.523^a^
Uric acid, mg/dl	7.71 ± 1.571^a^	7.79 ± 1.682^a^	6.69 ± 0.613^a^	6.85 ± 0.664^a^	7.18 ± 0.732^a^	6.55 ± 0.583^a^

*^a, b^Means within a row with different superscripts are significantly different (P < 0.05). SE, standard error. 1. corn soybean meal diet (Control +); 2. corn soybean meal diet + antibiotic (Control –); 3. corn soybean meal diet + 10% degraded date pits; 4. corn soybean meal diet + 0.2% mannan-oligosaccharides; 5. corn soybean meal diet + 0.2% Mannose; 6. corn soybean meal diet + 0.1% Mannose. Alanine aminotransferase (ALT), aspartate aminotransferase (AST), Gamma glutamyl transferase (GGT)*.

The supplementation of 10% DDP, 0.2% MOS, and 0.1% or 0.2% mannose in broiler diets had no significant effect on WBC, RBC, Hb, HCT, MCV, or MCHC. The MCH was significantly upregulated in broilers fed with the 10% DDP, 0.1% mannose, and 0.2% MOS diets. The Hb level, HCT, and MCHC were higher in broilers fed with the 10% DDP and 0.2% MOS diets but did not differ significantly from that in the other groups (Table 8).

**Table 8:** Effect of different dietary treatments on blood and serum hematological parameters of broilers (Means ± SE; *n* = 6).

**Parameter**	**Dietary treatments**					
	**1**	**2**	**3**	**4**	**5**	**6**
WBC, 10^3^/μl	21.9 ± 4.60^a^	22.3 ± 1.89^a^	24.2 ± 2.77^a^	24.9 ± 3.01^a^	21.5 ± 3.34^a^	24.1 ± 3.00^a^
RBC, 10^6^/μl	2.21 ± 0.561^a^	2.17 ± 0.600^a^	2.70 ± 0.770^a^	2.59 ± 0.661^a^	2.36 ± 0.471^a^	2.49 ± 0.512^a^
Hb, mg/100 ml	12.6 ± 1.04^a^	12.0 ± 0.85^a^	14.8 ± 1.30^a^	14.5 ± 1.11^a^	13.4 ± 1.18^a^	14.1 ± 1.04^a^
HCT, %	25.9 ± 3.01^a^	25.2 ± 2.63^a^	32.8 ± 4.26^a^	32.1 ± 3.99^a^	27.4 ± 3.34^a^	31.3 ± 3.68^a^
MCV, fl	135 ± 1.7^a^	132 ± 1.6^a^	138 ± 2.5^a^	138 ± 2.4^a^	135 ± 1.5^a^	137 ± 2.1^a^
MCH, pg	47.9 ± 0.71^b^	45.3 ± 0.48^b^	49.5 ± 0.70^a^	50.4 ± 0.75^a^	48.1 ± 0.66^b^	49.3 ± 0.88^a^
MCHC, %	49.2 ± 1.70^a^	47.4 ± 2.92^a^	53.3 ± 3.50^a^	50.4 ± 2.90^a^	48.2 ± 2.74^a^	53.8 ± 4.31^a^

*^a, b^Means within a row with different superscripts are significantly different (P < 0.05). SE, standard error; 1. corn soybean meal diet (Control +); 2. corn soybean meal diet + antibiotic (Control –); 3. corn soybean meal diet + 10% degraded date pits; 4. corn soybean meal diet + 0.2% mannan-oligosaccharides; 5. corn soybean meal diet + 0.2% Mannose; 6. corn soybean meal diet + 0.1% Mannose. WBC, white blood cells; RBC, red blood cells; Hb, hemoglobin, HCT, hematocrit value; MCV, mean corpuscular volume; MCH, mean corpuscular hemoglobin; MCHC, mean corpuscular hemoglobin concentration*.

## Discussion

The antioxidant properties of date pits degraded with *T. reesei* along with MOS content were considerably increased compared to NDDP. In addition, mannose, glucose, arabinose, rhamnose, and glucuronic acid were significantly increased in the MOS of DDP compared with NDDP. The present results indicate that degradation caused structural modification of date pits. Compared with NDDP, the total phenolic contents in DDP were significantly higher. Phenolic compounds have been used to exhibit a scavenging outcome against free radicals (33).

The nutrient content of food can be enhanced by microbial degradation through the biosynthesis of proteins, vitamins, and essential amino acids, which results in enhanced protein quality and fiber digestibility (34). Microbial degradation removes antinutritional factors and alters the bioavailability of micronutrients (35). During SSD, the catalytic action of β-glucosidase enhances phytochemical constituents such as flavonoids and phenolic compounds through the release of phenolic isoflavone aglycones, and the formation of reductases increases the antioxidant properties of legume seeds (36). The microorganisms involved in SSD cleave the phenolic and flavonoid linkages, which free the compounds to act as antioxidants, thereby improving antioxidant activity (37). The phenolic and flavonoid content of date pits significantly increased following degradation. DDP also showed significant antioxidant activity, as demonstrated by the results of the DPPH, ABTS, and FRAP assays. The increase in bioactive substances in DDP after treatment by *T. reesei* is of added value to agro-byproducts in view of improving the nutritive value as feedstuffs and extended their utilization as a growth promoter. Furthermore, after COVID-19 outbreaks, shortage in global feed resources is apparent due to strong competition between animals and humans for grains and cereals; thus, improving the utilization of locally available resources is urgent in the present circumstances (1–3).

The current study showed that the activity of CAT, SOD, and GPx in serum was significantly increased in broilers fed with the 10% DDP diet. This probably indicates that DDP enhanced the syntheses of antioxidant enzymes. Broilers are capable of adapting to oxidative stress by encouraging the synthesis of antioxidant enzymes. The antioxidant systems in the body have antioxidant enzymes such as SOD and GPx, which act to protect the body from oxidative stress. The dismutation of superoxide ions to hydrogen peroxide by SOD is usually the primary defense. SOD is widespread in oxygen metabolizing cells and protects aerobic cells against the harmful effects of superoxide radicals and other ROS (38).

The antioxidant enzymes SOD and GPx are essential elements of the first level of antioxidant defense in the cell because they form the main protective system against oxidative damage (39). The serum levels of GSH significantly increased (*P* < 0.05) in the DDP treatment, especially by the 21st day of the experiment (40). The methanolic extract of *P*. *dactylifera* pits is known as an antioxidant exporter of β-carotene and phenolic compounds (41). This antioxidant action was linked with the phenolic contents (42).

The liver controls numerous essential mechanisms in the body, being the major organ for detoxification of different substances. The pathogenesis of liver defects covers different cell types in the liver through cell death and regeneration mechanisms. Ferket et al. (43) studied the advantages of the addition of MOS to poultry feed and reported that MOS enhances liver morphology and functioning. Sarangi et al. (44) studied the effect of dietary supplementation of prebiotics, probiotics, and symbiotics on liver histomorphology in broilers and reported the beneficial effects of MOS. The inclusion of prebiotics and peppermint extract in broiler diet enhanced the performance, enzymatic activity, and histological aspects of the liver during the experimental period (45).

Dietary supplementation of 10% DDP and 0.2% MOS enhanced the activity of antioxidant enzymes in the liver. The scavenger functions of these hepatic antioxidative enzymes protect the liver from oxidative deterioration by inducing the release of SOD and GPx from prebiotics such as 10% DDP and 0.2% MOS. The MDA content was significantly (*P* < 0.05) reduced in broilers fed with the 10% DDP and 0.2% MOS diets. Binding free radicals to chelating transition metal ions and hydrogen atoms that act as a catalyst for free radical generation could be the major mechanism in the antiperoxidative capability of such prebiotics (46). Dvorska and Surai (47) reported that dietary supplementation of MOS decreased the MDA level in quail liver. It was also reported that carbohydrates and carbohydrate-containing molecules can be used as antioxidants that scavenge ROS (48). Several trials reported that MOS has a protective impact on heat-stressed birds (49) and enhanced the antioxidant function of egg yolk and antioxidant enzymes in the liver (50) when fed to laying hens. Oskoueian et al. (51) reported that an ethanolic extract of palm kernel cake (PKC) has high levels of fatty acids and many bioactive compounds with significant antioxidant activity.

Dietary treatment with 10% DDP and 0.2% MOS upregulated the antioxidant enzyme levels in the intestine of broilers. It was found (52) that MOS from *Saccharomyces cerevisiae* has antioxidative action *in vitro*. This indicates that MOS can prevent the gut not only by removing undesirable bacteria but also through improving antioxidant status. Prebiotics like β-glucans have antioxidant action and enhance antioxidant enzyme levels in the intestine of broilers (53). The activity of antioxidant enzymes depends on dietary antioxidants. Oxidative damage increases when the antioxidant/oxidant balance changes in a negative manner due to increasing oxidative stress (54).

The increase in GPx activity in broilers fed with the 10% DDP and 0.2% MOS diets suggests greater protection from oxidative stress. The present results further substantiate the positive effect of 10% DDP and 0.2% MOS on GPx activity in chicken serum, intestine, and liver. GPx is active fundamentally in the cytoplasm of the cells and only about 10% of its activity takes place in the mitochondria (55). The endogenous MDA level reflects lipid peroxidation. The MDA level decreased in broilers fed with the 10% DDP and 0.2% MOS diets, and this was a direct reflection of decreased peroxidation and an enhanced protective effect.

Dietary inclusion of 10% DDP and 0.2% MOS in broiler diets did not affect blood biochemical parameters. The measurement of AST, ALT, and GGT activities helps determine any functional liver damage in broilers before clinical symptoms develop (56). Any abnormality in the increase in serum levels of AST and ALT can reveal hepatocellular damage; thus, normal levels of AST were observed in groups on 10% DDP and 0.2% MOS. Al-Bowait and Al-Sultan (57) showed that date pits had no significant impact on the blood glucose level in broilers. Consistent with our observations, Masoudi et al. (58) demonstrated that replacement of corn by 30% date pits in broiler feed had no significant impact on blood cholesterol, triacylglycerol, HDL, and LDL. The inclusion of date waste at 150 g/kg in isocaloric, isonitrogenous broiler diets from 1 to 42 days of age did not affect growth performance or blood cholesterol (59). Toghyani et al. (60) reported that broilers supplemented with β-glucan prebiotics or corn-soy diet have normal levels of serum biochemical parameters. The MOS from yeast autolysate in the diet lowered serum total cholesterol and triglycerides but had no effect on total protein and uric acid (61). The present study also supports the above findings. Moreover, yeast culture supplementation with MOS had no influence on serum parameters, but increased serum uric acid (62).

The present study showed no considerable changes in the blood hematological parameters in all treatments despite a significant increase in MCH in broilers fed with the 10% DDP, 0.1% mannose, and 0.2% MOS diets. This is consistent with Cetin et al. (63), who found that the addition of prebiotic MOS to the feed significantly (*P* < 0.05) increased the erythrocyte count, hemoglobin concentration, and hematocrit rates in turkeys. The elevation in MCH concentration observed herein could be attributed to the increase in the RBC count.

In conclusion, *T. ressi* degradation of date pits increased the antioxidant properties of DDP by increasing the availability of their phenolic compounds. That was reflected in the liver and intestinal enzymes of broilers. Hence, DDP could be fed at 10% to broilers to enhance antioxidant balance, improve product quality, and extend the use of DDP as a feedstuff and growth promoter in animal nutrition. This is of vital importance under COVID-19 circumstances due to the global shortage in feed supply and the unseen future for the availability of feed resources for animal nutrition and the expected damages in agriculture sector.

## Data Availability Statement

The raw data supporting the conclusions of this article will be made available by the authors, without undue reservation.

## Ethics Statement

The scientific committee of the Department of Integrative Agriculture, College of Food and Agriculture, United Arab Emirates University approved the present experiment.

## Author Contributions

All authors listed have made a substantial, direct and intellectual contribution to the work, and approved it for publication.

## Conflict of Interest

The authors declare that the research was conducted in the absence of any commercial or financial relationships that could be construed as a potential conflict of interest.
